# A Comparative Prospective Study Between Conventional Chemo-Radiotherapy and Pure Accelerated Radiotherapy With Concurrent Chemotherapy for the Treatment of Locally Advanced Head and Neck Cancer

**DOI:** 10.7759/cureus.42206

**Published:** 2023-07-20

**Authors:** Sumana M Das, Niladri Roy, Dharmendra Singh, Pritam Kumar Sardar, Siddhartha Das

**Affiliations:** 1 Department of Radiotherapy, Radha Gobinda (RG) Kar Medical College and Hospital, Kolkata, IND; 2 Department of Radiotherapy, Medical College and Hospital, Kolkata, IND; 3 Department of Radiotherapy, All India Institute of Medical Sciences, Deoghar, Deoghar, IND; 4 Department of Radiotherapy, Malda Medical College and Hospital, Malda, IND; 5 Department of Physiology, Diamond Harbour Government Medical College, Diamond Harbour, IND

**Keywords:** acute side effects, dfs, pure accelerated radiotherapy, conventional chemoradiotherapy, locally advanced head neck cancer

## Abstract

Background: The established standard treatment for locally advanced head and neck squamous cell carcinoma is concurrent chemoradiotherapy, but the optimum radiotherapy schedule for best disease control and acceptable toxicity is still evolving. Tumor control probability decreases with each day's prolongation of treatment time. Shortening the overall treatment time of radiation by pure accelerated radiotherapy may be a good option.

Material and methods: One hundred and sixty-five patients with histopathologically proven squamous cell carcinoma of the head and neck were included in the study and were assigned into two groups from January 2017 to June 2019. The total dose of 70 Gy was given, 2 Gy/fraction/day. Treatment was given five days a week (conventional radiotherapy) and six days a week (pure accelerated radiotherapy). Both groups received weekly concurrent injections of cisplatin.

Results: The stage (p=0.006) and fractionation of radiation (p=0.018) were the independent factors affecting disease-free survival (DFS). There was a statistically significant difference (p=0.019) in the recurrence of patients in different fractionation schedules. The median DFS was 39 months with a 95% CI of 31.44 - 46.55. One- and three-year DFS was 51% and 8.5% respectively in the five fractions/week schedule arm while 54.5% and 9.5% respectively in the six fractions/week schedule group.

Conclusion: Pure accelerated radiotherapy is more efficacious in terms of disease control with comparable mildly increased acute side effects.

## Introduction

Head and neck cancers (HNCs) are malignancies of the upper aerodigestive tract. More than 90% of cases are squamous cell cancer [1]. Radiotherapy (RT) has long been the standard of care for locally advanced non-resectable diseases. According to MARCH meta-analysis, altered fractionated RT with reduced overall treatment time resulted in improved locoregional control (LRC) and overall survival (OS) compared to conventionally fractionated RT [2]. Types of altered fractionation were hyperfractionated accelerated radiotherapy (HA-RT) and accelerated fractionation with concomitant boost (AF-CB) [2,3].

RTOG 90-03, EORTC 22791, and DAHANCA are the landmark trials for the optimal radiation-alone protocol and used for guidance of the adequacy of the RT component of different chemo-radiation arms, but all these newer radiation protocols led to only 7 to 10% improvement in LRC [3-5], and no consensus also exists for the optimal radiation dose fractionation scheme.

The importance of overall treatment time (OTT) was illustrated dramatically in retrospective analysis by Overgaard in the DAHANCA trial. They tested pure acceleration (66 Gy/5.5weeks) against conventional RT (66 Gy/6.5 weeks) and showed the importance of OTT in local control of rapidly proliferating tumors like HNC in which the mean potential doubling time (T pot) is four days [5]. In the 1980s, Withers first showed that local control is lost if OTT is prolonged and approximately 0.6 Gy/day is required to compensate for accelerated repopulation of tumor tissue [6]. Irradiation given more intensively would be more effective at better tumor control.

However, the superiority of concurrent chemoradiotherapy (CRT) over RT alone was proven by MACH-NC meta-analysis, which included 3,727 patients of 63 different trials (conducted between 1965 and 1993) and showed an absolute survival benefit of 4% at five years favoring those who are on a combined modality arm [7]. An update by including another 24 trials revealed an 8% benefit in the five-year OS of CRT [8], and cisplatin-based therapy became the standard treatment for unresectable HNSCC [9]. After analyzing the Princess Margaret trial on concurrent HNC, it was stated differently that chemotherapy was equivalent to approximately 17 Gy of additional irradiation. An RTOG three-arm trial also clearly showed that concurrent therapy is the most effective means of larynx preservation and the best disease control but without survival benefit [10]. The superiority of CRT is also proven in squamous cell carcinoma of other anatomical sites like the cervix and esophagus [11,12]. Most institution refers to 100 mg/m^2 ^dosing of injecting cisplatin on day 1, 22, and 43 as standard. But frequent administration in lower doses may provide a radio-sensitizing effect during a larger proportion of the course of RT and less chemotherapy-induced morbidity without compromising efficacy [13].

So, an unresolved question is whether altered fractionation irradiation like accelerated irradiation and concurrent chemotherapy is superior to conventionally fractionated RT and CRT. In some studies, the feasibility of HA-RT or AF-CB plus chemotherapy was shown [14]. According to two randomized trials, accelerated RT plus chemotherapy did not result in improved outcomes [15,16].

## Materials and methods

This prospective comparative interventional study was carried out at the Department of Radiotherapy at IPGME&R, Kolkata, West Bengal, from January 2017 to June 2019. Prior to the commencement of the study, approval of the Institute Ethical Committee was obtained, and each subject was informed in detail of the study's objective, the aim of the research protocol, and the method to be used.

Each enrolled subject was assessed clinically by detailed examination including indirect laryngoscopy (I/L), direct laryngoscopy (D/L), nasopharyngoscopy, and pan-endoscopy. Contrast-enhanced computed tomography (CECT) face and neck or contrast-enhanced magnetic resonance imaging (CEMRI) was done to know the local extent of the disease, CECT thorax, and USG whole abdomen or CECT whole abdomen to rule out distant metastasis. Patients were staged according to American Joint Committee on Cancer (AJCC) 7th edition [17]. 

Patients included in the study had histopathology-proved squamous cell carcinoma of the head and neck excluding the nasopharynx, without any distant metastasis, aged from 18 to 70 years, and did not have any history of prior RT or chemotherapy, and all the blood biochemical parameters were within normal limits including creatinine clearance > 60 ml/min and Eastern cooperative oncology group (ECOG) performance status (PS) from 0-2 [18]. Creatinine clearance was measured by using the Cockcroft-Gault formula [19]. 

Patients with carcinoma other than squamous cell origin or with distant metastasis, pregnant woman, and those who did not sign the informed consent documents were excluded from the study.

Prior to the start of RT, pre-radiation dental or oral prophylaxis was done and a gap of minimum two weeks was given in case of tooth extraction. Patients were immobilized in a thermoplastic immobilization mask device with an appropriate headrest to make the spine straight. 2-D treatment planning was done with the help of a CT simulator. Treatment was given by telecobalt treatment unit Bhabatron II (Panacea Medical Technologies, headquartered in Bangalore, India). Two lateral parallel opposed fields were used to treat primary tumors and upper neck nodes, and a properly matched separate low anterior neck (LAN) field was used to treat the lower neck. Special care was taken not to place field junctions over gross disease. The off-cord technique was used after 46 Gy by shifting the posterior field border anterior to the spine to respect the spinal cord tolerance. Patients with residual disease in the posterior neck were treated accordingly.

Initially, 165 patients were divided into two groups by the lottery method. The first group (control) included 84 patients treated with five fractions per week (5fr/wk), and in the second (experimental) group, 81 patients were treated with six fractions per week (6fr/wk). Planned OTT was 6.5 weeks and 5.5 weeks in the two groups, respectively. The radiation dose was 70 Gy in 35 fractions. Both groups received concurrent weekly injections of cisplatin 40 milligrams per meter square. During radiation therapy, six and nine patients defaulted in the 5 fr/wk group and the 6 fr/wk group respectively as they discontinued treatments and could not be contacted further. Analysis was done with the remaining total of 150 patients: 78 and 72 patients in 5 fr/wk and 6 fr/wk treatment groups, respectively.

Injection cisplatin 40 milligrams per meter square was given as an intravenous infusion every Monday, one hour before radiation therapy with proper hydration and premedication. Patients were also evaluated every Monday with thorough clinical examination, and a review of hematological and biochemical parameters for assessment of acute radiation toxicity according to the RTOG acute radiation toxicity assessment criteria was done. 

After completion of RT, patients were assessed every six weeks clinically and radiologically if necessary to assess tumor response and early recurrence. Disease-free survival (DFS) was defined as the time from potentially curative treatment until the recurrence of the cancer clinically. 

Statistical analysis

IBM SPSS Statistics for Windows, Version 25 (Released 2017; IBM Corp., Armonk, New York, United States) was used for the statistical analysis. Descriptive statistics were used to characterize the patient population. Continuous variables were analyzed using Student’s t-test. Univariate and multivariate analysis was done using logistic regression analysis. The factors found statistically significant in univariate analysis were included in multivariate analysis for survival outcomes. The Kaplan-Meier method was used to calculate and compare DFS. A p-value less than 0.05 is considered statistically significant in all the performed analyses.

## Results

Epidemiological characteristics

**Figure 1:**
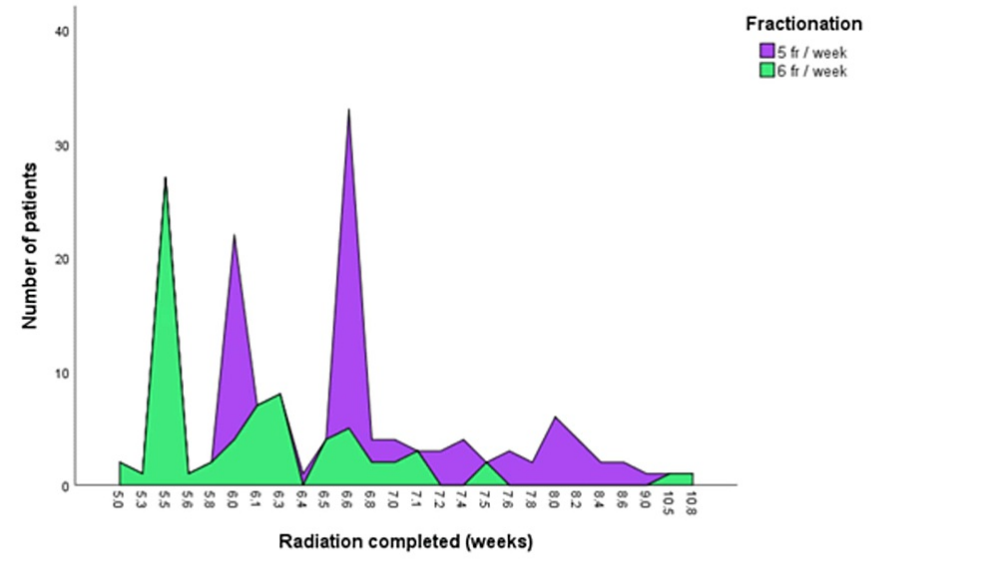
Duration of completion of radiation in weeks and the respective number of patients represented in two different fractionation schedules.

**Figure 2:**
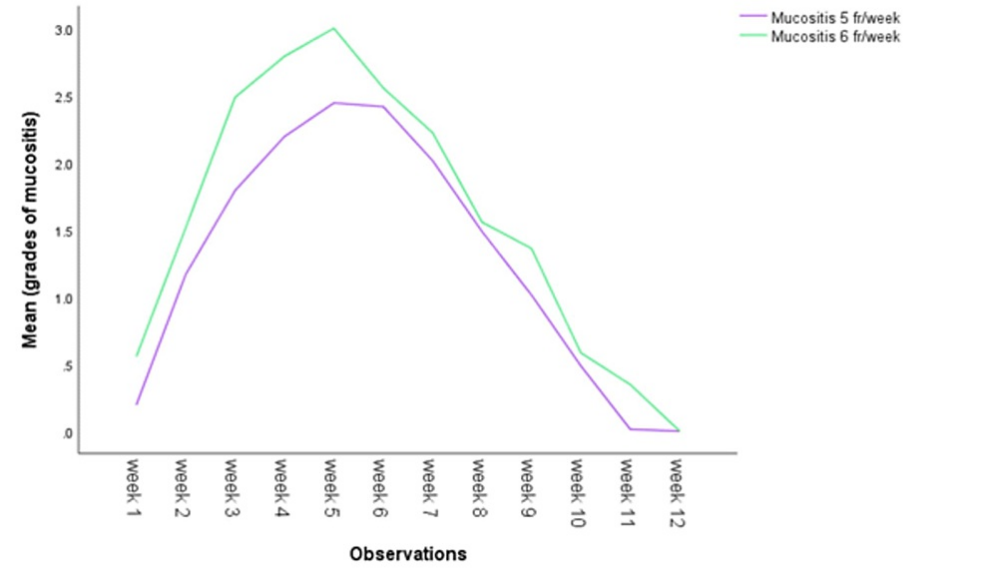
Grades of oral mucositis during the treatment and follow-up till complete recovery of mucositis in two different fractionation schedules

In this prospective comparative interventional study, there were 122 males and 28 females. The median age at diagnosis was 55 years. In both the groups, the most commonly affected age group was 45-55 years. There was no statistically (p=0.412) significant difference in the distribution of patients according to age group distribution. The overall most common primary site was the oral cavity with 35.3% followed by the oropharynx with 32%. There was no significant statistical difference in the distribution of patients in both the treatment groups (p=0.309). There was a history of addiction in 84% of patients. Smoking was the most common addiction and observed in 58.7% of patients followed by chewing tobacco in any form in 15.3% of patients. Stage III, stage IVa, and stage IVb were 55.9, 35.3, and 8.7% respectively. Histopathologically, well-differentiated, moderately differentiated, and poorly differentiated patients were 18.7%, 62.6%, and 18.7% respectively. In the classification of tumors according to the “T” group, we found that 32% of patients were in T2 and T3 groups while 66% were in T3 and T4 groups. Cervical neck nodes were positive for metastasis in 95.3% and only 4.7% of patients were negative for cervical neck node metastasis. During a median follow-up of 18 months, 44.7% of patients had recurrence. Detailed clinicopathological characteristics are depicted in Table 1. There was a statistically significant difference (p=0.019) of recurrence in both the treatment groups. The median duration of completion of radiation in the 5 fr/wk and 6 fr/wk schedule arms was 6.6 weeks and 6 weeks, respectively (Figure 1). The median prescribed dose was 70 Gy in both the radiation schedules. Mucositis of grade 3 was observed in 9.1% and 25.5% of 5 fr/wk and 6 fr/wk schedules, respectively (p<0.001) (Figure 2). Different grades of xerostomia in different schedules of radiation are given in Table 2. 

**Table 1:** Clinicopathological characteristics HTN: Hypertension; DM: diabetes mellitus

		Fractionation				P-value
		5 fractions / week		6 fractions / week		
		Count	N %	Count	N %	
Age group	< 45 years	10	12.82%	9	12.50%	0.412
	45 - 55 years	29	37.17%	28	38.88%	
	56 - 65 years	24	30.77%	27	37.51%	
	> 65 years	15	19.24%	8	11.11%	
Gender	Male	60	76.93%	62	86.11%	0.149
	Female	18	23.07%	10	13.89%	
Head and neck cancer site	Oral cavity	32	41.03%	21	29.16%	0.309
	Oropharynx	23	29.49%	25	34.73%	
	Hypopharynx	4	5.13%	5	6.95%	
	Larynx	8	10.25%	6	8.33%	
	Supraglottic larynx	11	14.10%	15	20.83%	
Addiction	None	12	15.30%	12	16.67%	0.910
	Smoking	46	58.99%	42	58.33%	
	Alcohol	2	2.58%	1	1.38%	
	Smoking and alcohol	5	6.45%	7	9.72%	
	Gutka / tobacco chewing	13	16.68%	10	13.90%	
Comorbidity	None	52	66.68%	59	81.95%	0.071
	Hypertension	17	21.80%	4	5.56%	
	DM	5	6.40%	5	6.96%	
	HTN and DM	1	1.28%	1	1.38%	
	Tuberculosis	1	1.28%	2	2.77%	
	Hypothyroidism	2	2.56%	0	0.00%	
	Seizure disorder	0	0.00%	1	1.38%	
Tumor group	T2	21	26.92%	20	27.77%	0.907
	T3 & T4	57	73.08%	52	72.23%	
T-size	T2	21	26.92%	20	27.80%	0.994
	T3	37	47.43%	35	48.60%	
	T4a	14	17.95%	12	16.66%	
	T4b	6	7.70%	5	6.94%	
N-size	N0	4	5.12%	3	4.16%	0.966
	N1	43	55.12%	39	54.16%	
	N2	26	33.34%	24	33.34%	
	N3	5	6.42%	6	8.34%	
Stage group	Stage II	0	0	0	0	0.867
	Stage III	45	57.69%	39	54.16%	
	Stage IVA	27	34.61%	26	36.11%	
	Stage IVB	6	7.70%	7	9.73%	
Histopathology	Well differentiated	16	20.51%	12	16.66%	0.223
	Moderately differentiated	44	56.42%	50	69.45%	
	Poorly differentiated	18	23.07%	10	13.89%	
Recurrence	Yes	42	53.84%	25	34.72%	0.019
	No	36	46.16%	47	65.28%	

**Table 2:** Grades of xerostomia in two different fractionation schedules

Grades of xerostomia in two different fractionation schedules				
		Fractionation		p-value
		5 Fractions /week	6 Fractions /week	
		Count	Count	
Week 1	Grade 0	63	55	0.513
	Grade 1	15	17	
	Grade 2	0	0	
	Grade 3	0	0	
Week 2	Grade 0	25	13	0.049
	Grade 1	53	59	
	Grade 2	0	0	
	Grade 3	0	0	
Week 3	Grade 0	2	0	0.005
	Grade 1	67	49	
	Grade 2	9	23	
	Grade 3	0	0	
Week 4	Grade 0	0	0	0.226
	Grade 1	38	28	
	Grade 2	40	44	
	Grade 3	0	0	
Week 5	Grade 0	0	0	0.658
	Grade 1	13	14	
	Grade 2	65	58	
	Grade 3	0	0	
Week 6	Grade 0	0	0	0.002
	Grade 1	7	0	
	Grade 2	58	46	
	Grade 3	13	26	
Week 7	Grade 0	0	0	0.001
	Grade 1	27	2	
	Grade 2	41	47	
	Grade 3	10	23	
Week 8	Grade 0	0	0	0.001
	Grade 1	28	9	
	Grade 2	42	44	
	Grade 3	8	19	
Week 9	Grade 0	0	0	0.014
	Grade 1	51	30	
	Grade 2	23	36	
	Grade 3	4	6	
Week 10	Grade 0	0	0	0.328
	Grade 1	59	52	
	Grade 2	19	18	
	Grade 3	0	2	
Week 11	Grade 0	0	0	0.665
	Grade 1	68	61	
	Grade 2	10	11	
	Grade 3	0	0	
Week 12	Grade 0	0	0	0.651
	Grade 1	63	56	
	Grade 2	15	16	
	Grade 3	0	0	

Correlation studies

We found that recurrence was statistically significantly correlated with the tumor size (p<0.001), cervical neck node metastasis (p<0.001), stage at diagnosis (p<0.001), and histopathological differentiation of the tumor or grade of the tumor (p=0.016). Recurrence was not significantly correlated with gender (p=0.583), the site of primary HNC (p=0.403), and addiction (p=0.160).

Prognostic factors affecting DFS

Logistic regression univariate analysis of the patients showed that T stage (HR 1.811; 95% CI 1.256-2.610; p=0.001), N stage (HR 1.991; 95% CI 1.464-2.707; p<0.001), stage group (HR 2.650; 95% CI 1.886-3.723; p<0.001), tissue differentiation (HR 1.507; 95% CI 1.028-2.209; p=0.036), and fractionation of radiation (HR 0.593; 95% CI 0.360-0.976; p=0.040) affect the DFS. Logistic regression multivariate analysis showed that stage (HR 3.159; 95% CI 1.397-7.140; p=0.006) and fractionation of radiation (HR 0.544; 95% CI 0.329-0.899; p=0.018) were the independent factors affecting the DFS (Table 3).

**Table 3:** Univariate and multivariate analysis of factors associated with disease-free survival HNC: Head and neck cancer; HR: hazard ratio; CI: confidence interval

Clinical characteristics	Univariate analysis			Multivariate analysis		
	HR	95% CI	p-value	HR	95% CI	p-value
Age group	0.906	0.698 – 1.177	0.461			
Gender	1.180	0.652 – 2.135	0.583			
T-size	1.811	1.256 – 2.610	0.001	1.416	1.005 – 1.994	0.047
N- size	1.991	1.464 – 2.707	< 0.001	0.852	0.440 – 1.652	0.636
Addiction	1.138	0.950 – 1.363	0.160			
Stage	2.650	1.886 – 3.723	< 0.001	3.159	1.397 – 7.140	0.006
Tissue differentiation	1.507	1.028 – 2.209	0.036	1.175	0.791 – 1.744	0.424
Site of primary HNC	0.946	0.830 – 1.078	0.403			
Fractionation	0.593	0.360 – 0.976	0.040	0.544	0.329 – 0.899	0.018

Survival analysis

**Figure 3:**
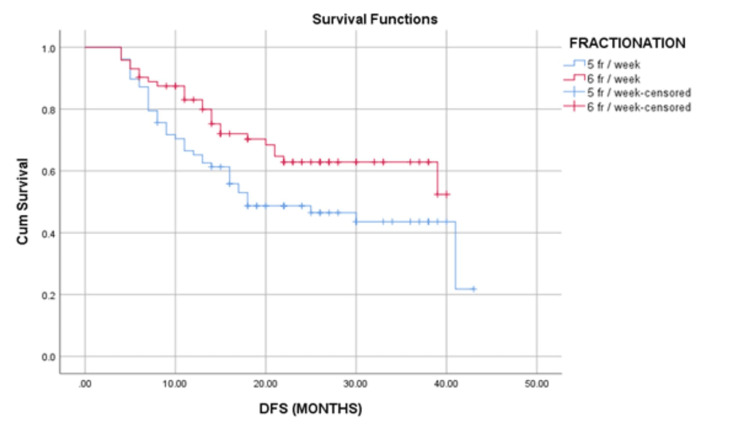
The Kaplan Meier survival curve according to different fractionation schedules. The median DFS was not reached in six fractions/week schedule. The median DFS was 18 months in five fractions/week schedule. The difference in DFS was statistically significant (P=0.035) DFS: Disease-free survival

The median follow-up duration was 18 (range 14 - 43) months. A total of 67 (44.7%) patients had recurrence. There was a statistically significant difference (p=0.019) in the recurrence of patients in different fractionation schedules. There was 53.84% and 34.72% recurrence in 5 fr/wk and 6 fr/wk groups, respectively. The median DFS was 18 months with a 95% CI of 5.078-30.922 in 5 fr/wk schedule arm. The median DFS was not reached in the 6 fr/wk schedule arm (p=0.035). The Kaplan-Meier survival curve of both groups is depicted in Figure 3. One- and three-year DFS was 51% and 8.5%, respectively, in the 5 fr/wk schedule arm while one- and three-year DFS was 54.5% and 9.5%, respectively, in the 6 fr/wk schedule arm.

## Discussion

The present study was designed to assess the efficacy and tolerance of pure accelerated radiation with concurrent chemotherapy. Our epidemiological findings matched with other Indian and international studies where 80% of head-neck malignancies occur within 45-65 years of age with a male-to-female ratio of 4:1 [20,21]. In India, among patients diagnosed with HNC, 86.5% were reported as tobacco users and 23.2% were reported as alcohol users [22]. Tobacco and alcohol frequently coexist and lead to adverse prognosis. Five-year OS in patients who consumed both alcohol and tobacco was 29% [23]. Our study also reflects a similar prevalence of addiction (84%) in any form either smoking or chewing tobacco.

Oral/mouth cancers were the second most common of all cancers and the most common HNC among males in the Indian subcontinent [24]. It may be due to the widespread habit of using tobacco in different forms. Another study showed that oropharyngeal carcinoma constitutes the maximum proportion. However, the oropharynx and oral cavity constituted the major burden of total head and neck cancer [21]. The majority (78%) presented with the inoperable stage as in another study where 66.6% presented in the advanced stage [23]. The maximum number of patients (48.7%) are of stage III followed by stage IVa (39.3%), whereas stage IV patients were the maximum in other studies [20,21]. Advanced stage due to delayed diagnosis because of varied reasons invariably led to a poor outcome as seen in other studies where the five-year median OS varied from 100% at stage I to 42% at stage IV [24]. The incidence of co-morbidities (hypertension) is much less (26%) in our study compared to other Indian studies by Tata Memorial Hospital, where it was about 57% [25].

T stage, N stage, grade, and the fractionation schedule influence DFS individually in our study. Among them, stage and fractionation are the two most important factors. No association with age or sex was found in our study. The importance of T size as a determinant of DFS was also proved in another study, but they did not find any correlation with tumor grade. Rather, they showed a significantly better prognosis in females in terms of both local control and survival [26]. However, studies also showed that advanced stage, male sex, older age, smoking, and alcohol were associated with the worst outcome [20]. 

As we all know from MARCH meta-analysis, altered fractionation RT mainly hyperfractionation was associated with a small but significant improvement (8.1% at five years) in OS compared with standard fractionation, but this benefit decreased with older age and when follow-up was censored at five years [2]. Hyperfractionation provides a major benefit on local control with a smaller benefit on nodal control and cancer mortality, but accelerated regimens only provided an improvement in local disease, not nodal disease. Therefore, pure acceleration should only be considered for patients with a low nodal burden [5].

Based on the results of the Danish protocols, it was clear that the RT regimen should be of the shortest possible overall treatment time to prevent accelerated repopulation. Furthermore, treatment with six fractions per week is now the standard RT in Denmark for most head and neck carcinomas, along with hypoxic radiosensitizer nimorazole. The response to accelerated fractionation is however heterogeneous.

DAHANCA, The Danish Head and Neck Cancer Group, has gone through a series of prospective trials for HNSCC. In the 1980s, the effectiveness of hypoxic radiosensitizer nimorazole was proved in LRC along with conventionally fractionated RT [27]. In the next decade, DAHANCA 6 & 7 trials showed a further increase in local control by one-week shortening of overall treatment time with 6 fr/wk week as a moderate acceleration with the same radiation dose [5]. One thousand four hundred and seventy-six eligible patients were divided into five (n=726) or six (n=750) fractions per week (except T1 glottic tumors) along with hypoxic radiosensitizer nimorazole. Median overall treatment times were 46 days in the 5fr/wk group and 39 days in 6 fr/wk groups. The five-year LRC was 70% and 60% for the 6 fr/wk and 5 fr/wk groups, respectively (p=0.0005). The whole benefit of treatment time reduction was evident for local control (76 vs 64% for 6 and 5 fractions per week, p=0.0001), but was not significant for neck-node control. Improved voice preservation is also seen in the accelerated group. DFS also improved (73% vs 66% for 6 and 5 fractions per week, p=0.01) but not OS. The acute reaction was significantly higher 6 fr/wk group but was transient [5]. After that, the DAHANCA 9 study explored further dose escalation to 76 Gy through hyperfractionation [28] and DAHANCA 18 study evaluated the efficacy of weekly concurrent chemotherapy and nimorazole to moderately accelerated RT by normo-fractionated IMRT in patients with locally advanced HNSCC respectively. A total of 227 patients (male: female; 4:1) with a median age of 57 years were analyzed. The five-year LRC, EFS, and OS were 80%, 67%, and 72%, respectively. It showed a positive result in node-positive patients.

Another Dutch study also tested the efficacy of pure acceleration (6 fr/wk) with weekly cisplatin vs conventional concurrent radiation with three weekly injections of cisplatin, but they had given four cycles of TPF as neoadjuvant (NACT) [29]. At a median follow-up period of 2.8 years, the trial was terminated prematurely by the data safety monitoring board. They showed lower grade 3-4 mucositis (22% vs 57%) and better two years PFS (72% vs 70%) and OS (79% vs 78%) in the accelerated arm.

We found a similar average duration of treatment and incidence of mucositis, but DFS differs much from the previous studies [5]. The reason for this may depend on the method of radiation therapy (2D, 3D-CRT, or IMRT) and treatment equipment (Telecobalt or linear accelerator). So, these patients must require more aggressive treatment. This fact is reflected to some extent in this study. Patients in the accelerated arm showed relatively better DFS than the control arm. 

There were also many Indian and international studies that compared pure accelerated RT alone with conventional CRT. In a three-arm trial, Patil et al. showed better locoregional disease control (49%; p = .049) with CRT over pure accelerated RT alone but later showed higher but acceptable acute and late toxicities [22]. Gupta et al. also showed better one-year DFS in CRT than pure accelerated RT alone (70.1% vs.62.1% P = 0.296,) with statistically significant increased grade 4 toxicities in the CRT arm (32.8% vs 12.1%; P = 0.02) [20]. Our study shows better one-year DFS (54%) with addition of chemotherapy with pure accelerated RT.

The International Atomic Energy Agency (IAEA) ACC trial showed better five-years LRC (42%vs 30%) (HR 0.63, 95% CI 0.49-0.83; p=0.004) in accelerated RT alone compared with conventional RT alone with more severe acute skin reaction without any effect on late toxicity in the accelerated group. So, they concluded that an accelerated schedule of RT for HNSCC was more effective than conventional fractionation. 

GORTEC 99-02, a three-arm open-label randomized study, did not show any promising outcome in accelerated RT arms over conventional chemo RT arms [15]. After a median follow-up of 5.2 years, they showed that accelerated RT-chemotherapy offered no PFS benefit compared with conventional chemoradiotherapy (HR 1·02, 95% CI 0·84-1·23; p=0·88) or very accelerated RT (0·83, 0·69-1·01; p=0·060); conventional chemoradiotherapy improved PFS compared with very accelerated RT (0·82, 0·67-0·99; p=0·041). More patients in the very accelerated RT group had RTOG grade 3-4 acute mucosal toxicity (226 [84%] of 268 patients) compared with accelerated RT-chemotherapy (205 [76%] of 271 patients) or conventional chemoradiotherapy (180 [69%] of 262; p=0·0001). They concluded that acceleration of RT is probably not beneficial in concomitant chemoradiotherapy schedules. but in our study, the outcome is quite impressive in terms of DFS with less toxicity.

RTOG 0129 a phase III Trial of cisplatin plus an accelerated concomitant boost versus standard fractionation analyzed 721 for OS, progression-free survival, and locoregional progression and concluded that the RT duration and cisplatin dose affected survival significantly. In our study, we also found a similar result.

Units for chemotherapy administered were taken from the study by Gupta et al. [30].

## Conclusions

Pure accelerated radiation with concurrent chemotherapy is an excellent option to achieve better tumor control and a reproducible result in locally advanced head and neck carcinoma with a mild increase in acute toxicities. This study showed the efficacy of this treatment regimen as a suitable alternative in the teletherapy machine (Bhabatron II treatment unit with planning software ONCENTRA version 4.5.2.23). However, with more sophisticated techniques like IMRT in LINAC, it may be possible to improve DFS further as well as lessen the toxicities. So, further randomized controlled trials with a large sample size will be needed for better understanding of the effectiveness of this treatment regimen.

Most of the HNCs are treated with modern LINAC or photon uses 6 MV energy which is equivalent to energy Telecobalt therapy unit. In a resource-constraint country with a long waiting list for radiation therapy, our study is relevant while considering the limited resources and reducing the long waiting list for radiation therapy as radiation oncology is still evolving day by day but findings from different studies done years back cannot be ignored. The benefit of concurrent chemo-radiation in HNC is mostly cited from meta-analyses such as MACHNC. In this meta-analysis, most of the studies included the radiation techniques of 3DCRT with LINAC energy of 6MV. In this regard, our study is still equivalent in terms of use of energy. We have seen increased mucositis in our study due to the technique of radiation.

The only problem with cobalt is that we have to use 2D or 3D techniques, while in LINAC, we use IMRT and VMAT to spare our normal tissues. But again the use of CTRT is still on the basis of meta-analysis; there were very few studies that used IMRT. The use of proton therapy in HNCs is still under trial and results of most of the studies are awaited and the availability of proton therapy is very limited.
